# Precise management of thick skin flaps after mandibular fibula muscle flap transplantation and implant restoration: a case report

**DOI:** 10.3389/froh.2025.1704452

**Published:** 2025-12-01

**Authors:** Zhen Ci, Jingxia Chen, Jiaxin Luo, Chuqiao Wei, Jian Feng, Hanchi Wang, Yanmin Zhou

**Affiliations:** 1 Department of Oral Implantology, Hospital of Stomatology, Jilin University, Changchun, China; 2 Jilin Provincial Key Laboratory of Tooth Development and Bone Remodeling, Hospital of Stomatology, Jilin University, Changchun, China

**Keywords:** dental implant, mandibular fibular graft, myocutaneous flap, mandibular reconstruction, custom healing abutment, soft tissue management

## Abstract

Implant-supported restorations are critical for rehabilitating jawbone defects and restoring patients' occlusal function. Achieving proper fit and occlusal relationship with opposing teeth, as well as establishing a healthy peri-implant soft tissue seal, is essential to prevent infection and promote maintainability. This article presents a case involving the precise management of a thick soft tissue flap and the placement of an implant-supported restoration following mandibular reconstruction with a fibula flap. It offers a comprehensive overview of the diagnostic approach, surgical procedures, and prognostic outcomes, with the aim of providing valuable insights for clinical practice. The review spans the entire treatment process—from the planning of Stage I implant surgery, including optimal implant positioning and type selection, to the intraoperative techniques for layered debulking and flap preconditioning. It further discusses the evaluation of soft tissue thickness and the application of a customized healing abutment during Stage II surgery, as well as strategies for long-term oral hygiene maintenance.

## 1 Introduction

Jawbone defects resulting from the resection of maxillofacial tumors not only cause facial deformities but also significantly impair or even completely compromise masticatory function (1). Surgical reconstruction of the maxilla restores its continuity and helps preserve the patient's normal facial contour. With implant-supported prosthetic rehabilitation, the dentition can be functionally reconstructed, thereby restoring chewing capacity and achieving true functional reconstruction of the jaw (2). The combination of bone grafting and dental implantation represents an ideal approach for the repair of mandibular defects. Successful outcomes include complete viability of the free vascularized fibula graft, stable implant fixation, satisfactory restoration of masticatory function, improved facial aesthetics, and absence of significant complications. However, anatomical alterations, discrepancies in vertical bone height, and excessive soft tissue thickness often reduce the elasticity of the transplanted flap, promoting plaque accumulation and increasing the risk of peri-implantitis in the long term. Thus, optimized soft tissue management is critical for preventing peri-implant inflammation (3). Following prosthetic restoration, patients must adhere to regular follow-up visits and maintain appropriate oral hygiene practices, both of which are essential for the long-term success of the implant-supported reconstruction.

## 2 Case presentation

### 2.1 Basic information

The patient is a 49-year-old female who underwent “mandibular tumor resection followed by titanium plate reconstruction” nine years ago. She presents with a missing mandibular dentition and desires implant-supported restoration.

**Figure 1:**
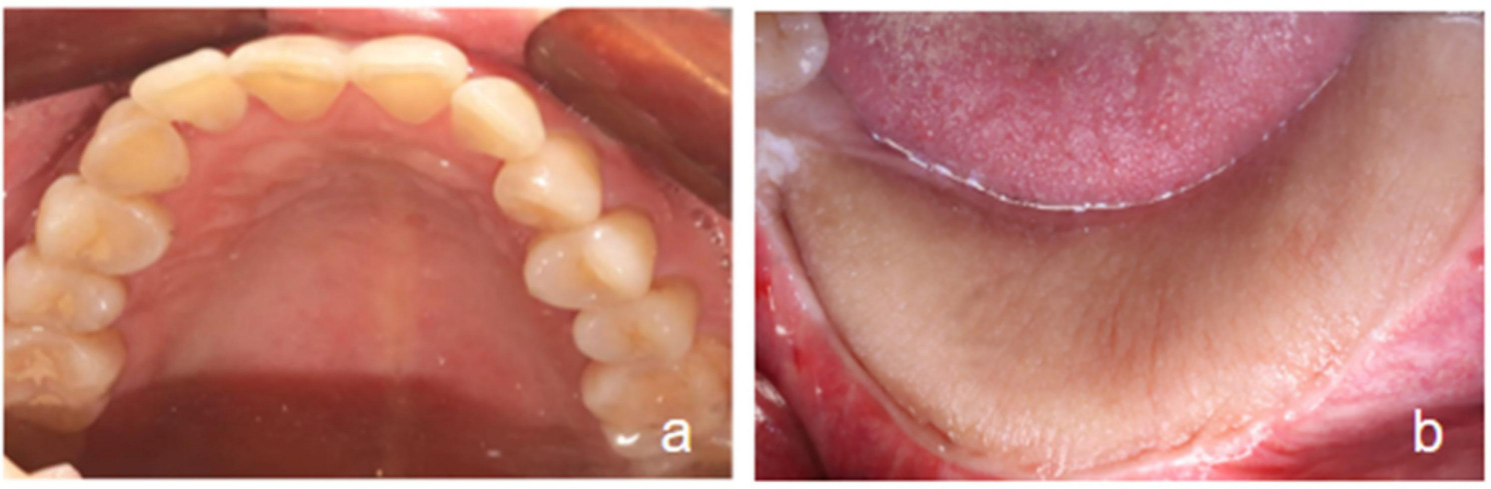
Intraoral examination. **(a)** Complete upper dental arch; **(b)** Teeth #37 to #46 are missing, with the presence of a large intraoral skin flap graft in the edentulous region.

**Figure 2:**
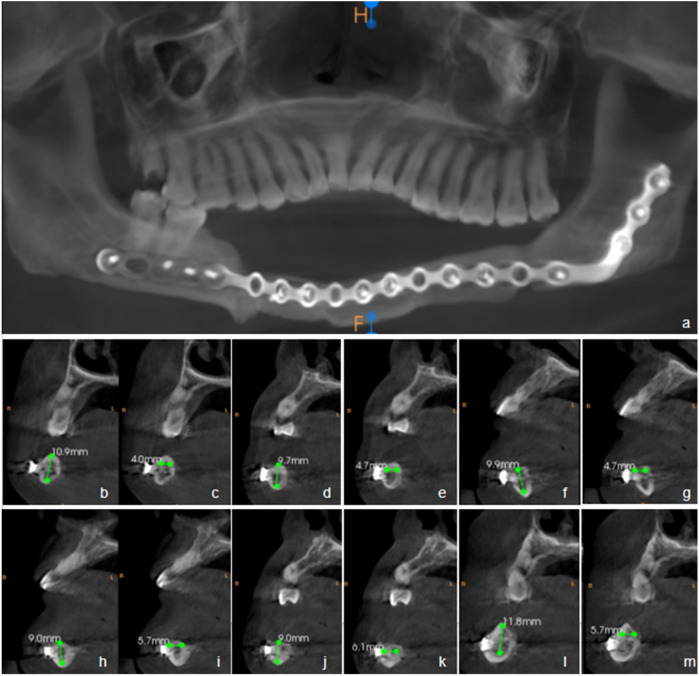
Preoperative cone-beam computed tomography (CBCT) imaging and implant surgical planning. **(a)** Panoramic reconstructed view; **(b–m)** Serial cross-sectional images of planned implant positions; **(b)** Bone height at implant site #36; **(c)** Buccolingual ridge width at site #36 (measured 1 mm apical to the crest).(using the level 1 mm below the alveolar ridge crest as the baseline). **(d)** Bone height at implant site #35; e. Buccolingual ridge width at site #35 (measured 1 mm apical to the crest); **(f)** Bone height at implant site #32; g. Buccolingual ridge width at site #32 (measured 1 mm apical to the crest); **(h)** Bone height at implant site #42; i. Buccolingual ridge width at site #42 (measured 1 mm apical to the crest); **(j)** Bone height at implant site #45; k. Buccolingual ridge width at site #45 (measured 1 mm apical to the crest); **(l)** Bone height at implant site #46; **(m)** Buccolingual ridge width at site #46 (measured 1 mm apical to the crest).

(1) Oral Examination: Teeth #37 to #46 are missing. The edentulous alveolar ridge exhibits insufficient vertical height. A large transplanted flap is observed in the corresponding region. Oral hygiene is satisfactory (Figure 1).
(2) Imaging Examination: Cone-beam computed tomography (CBCT; NewTom VGI, QR Srl, Verona, Italy) confirms the absence of teeth #37 to #46. The bony union between the fibular graft and the native mandible is well consolidated. The reconstruction titanium plate and screws are in a stable position. The alveolar bone in the edentulous region (#37–46) demonstrates adequate bone height and width to meet the criteria for delayed implant placement (Figure 2).

### 2.2 Treatment plan

Preoperative cone-beam computed tomography (CBCT) was used to evaluate bone volume and to localize the existing reconstruction titanium plate and screws. Based on the CBCT findings, an optimal implant placement scheme was designed. A standard delayed implant protocol was subsequently carried out. Four months postoperatively, an impression was made, followed by the fabrication and delivery of the implant-supported prosthesis.

### 2.3 Surgical and restorative procedures

#### 2.3.1 First-Stage implant surgery

**Figure 3:**
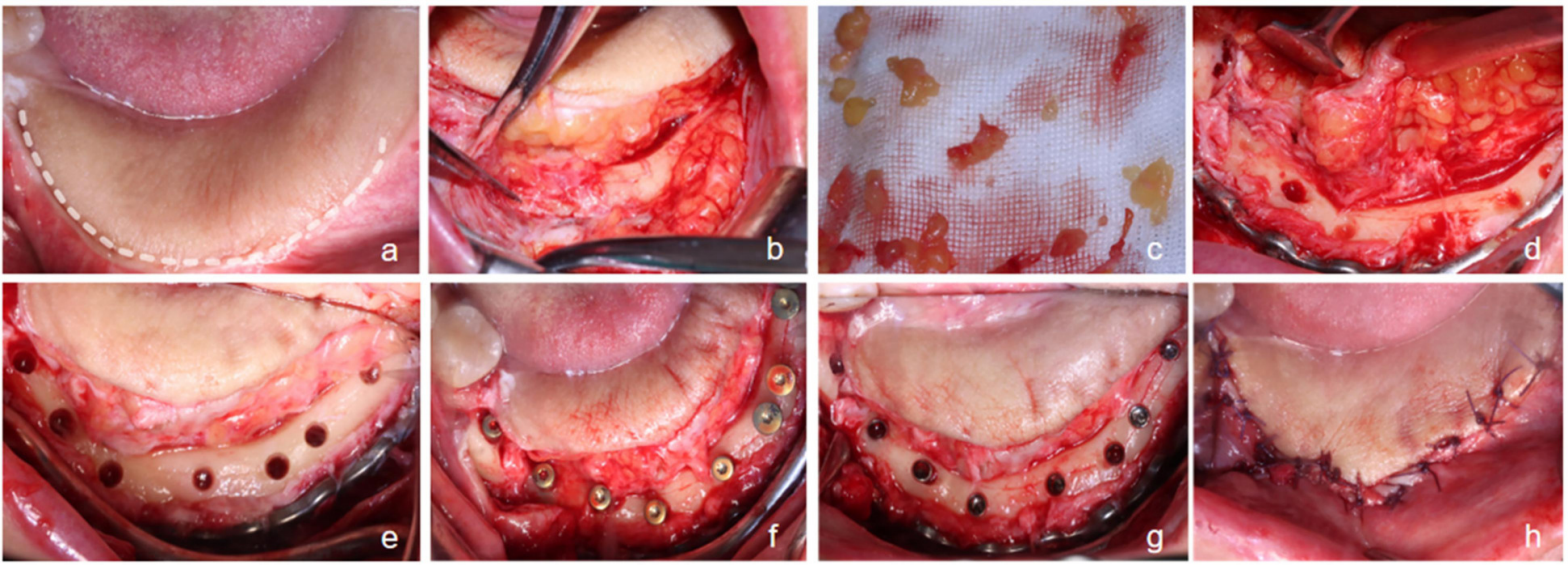
Stage 1 implant surgery process. **(a)** Incision design; **(b)** Incision along the gingiva-flap junction and periosteal flap elevation; **(c)** Partial superficial adipose tissue excision during flap reflection; **(d)** Full exposure of the alveolar ridge crest and titanium plate/screw positions, followed by pilot drill guidance; **(e)** Osteotomy preparation at eight implant sites; **(f)** Implant placement; **(g)** Connection of non-submerged healing abutments; **(h)** Wound closure with meticulous suturing.

Following the provision of informed consent, the patient was thoroughly advised of the potential risks, procedural details, and expected recovery course. Upon agreeing to the treatment plan, the patient proceeded to surgery. Standard preoperative disinfection and sterile draping were performed. Local anesthesia was achieved using infiltration of 4% articaine hydrochloride with epinephrine 1:100,000.A horizontal incision was made along the crestal aspect of the alveolar ridge at the edentulous site (#37–46), following the junction between the native gingiva and the grafted flap. This was supplemented by intrasulcular incisions at the mesial adjacent teeth and two vertical releasing incisions. A full-thickness flap was elevated to expose the alveolar ridge crest, the buccal aspect of the reconstruction titanium plate, and the associated screws.The superficial portion of the flap was noted to consist of a 2–3 mm thick adipose layer. This fatty tissue was sharply dissected from the underlying muscle using a surgical blade. Approximately 50% of the superficial fat was excised, while carefully preserving the deep adipose layer containing the essential vascular network. The vertical releasing incisions were terminated approximately 1 mm apical to the deepest point of the existing vestibular sulcus. A submucosal dissection was carried out to advance the buccal mucosal flap, surgically deepening the vestibular sulcus by approximately 5 mm.Using preoperative CBCT data for surgical guidance, the optimal implant positions were identified. Osteotomies were prepared following a sequential drilling protocol under irrigation. Eight Straumann® Bone Level Tapered Roxolid® SLA implants (3.3 mm × 8 mm) were placed, each achieving an insertion torque of at least 35 N·cm. Healing abutments were connected, and wound closure was accomplished using 3-0 PDS® (Polydioxanone) sutures (Ethicon, Somerville, NJ, USA) (Figures 3a–h).

#### 2.3.2 Postsurgical care

**Figure 4:**
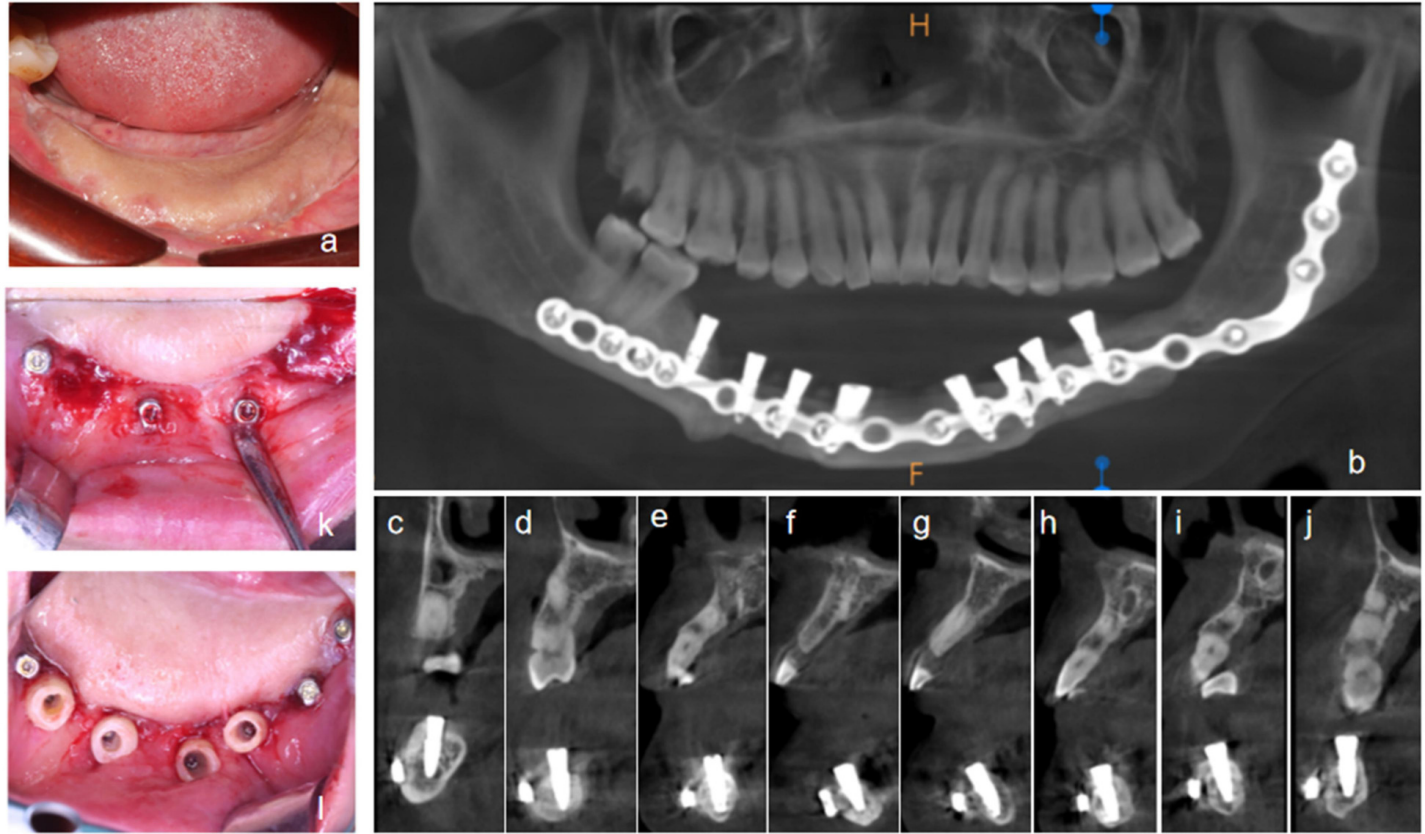
Second stage of implantation. **(a)** Clinical view at 18 days postoperatively after suture removal, showing well-healed soft tissue; **(b)** Panoramic reconstruction from CBCT scan taken four months after implant placement; **(c–j)** Sagittal views demonstrating successful osseointegration and proper positional relationship to the existing titanium reconstruction screws for all eight implants; **(k,l)** Placement of customized healing abutments during second-stage surgery.

The patient was prescribed a postoperative anti-inflammatory regimen consisting of intravenous ceftriaxone sodium (0.5 g twice daily) for three days. Additionally, oral diclofenac sodium (50 mg twice daily as needed) was provided for analgesia. The patient was instructed to rinse with 0.12% chlorhexidine gluconate mouthwash three times daily for two weeks. An 18-day follow-up examination revealed satisfactory healing of the surgical site, with no significant signs of redness or swelling. The mucosal tissues exhibited good integrity and healing progression (Figure 4a).

#### 2.3.3 Second phase of planting surgery

Four months after implant placement, the patient returned for a follow-up appointment. A cone-beam computed tomography (CBCT) scan was obtained (Figures 4b–j), which revealed no peri-implant radiolucency, indicating satisfactory osseointegration and permitting progression to the second-stage surgery. Soft tissue thickness measurements showed 9 mm in the anterior mandible and 7 mm in the posterior mandible. Following standard disinfection, sterile draping, and local infiltration anesthesia, the existing healing abutment was removed via a minimally invasive approach. A prefabricated, customized, and elevated healing abutment—fabricated by layering self-curing resin onto the original abutment followed by precision milling and polishing—was placed. The customized component had been stored in 0.12% chlorhexidine solution until installation. At the 10-day postoperative follow-up, the peri-abutment soft tissue exhibited close adaptation without gaps or bleeding on probing (Figures 4k, l). Implant stability was assessed using resonance frequency analysis (Osstell® ISQ, Osstell AB, Sweden), with all eight implants exhibiting implant stability quotient (ISQ) values greater than 75, fulfilling the criteria for prosthetic restoration. Final digital impressions were then made to proceed with the definitive crown fabrication.

#### 2.3.4 Upper crown restoration

**Figure 5:**
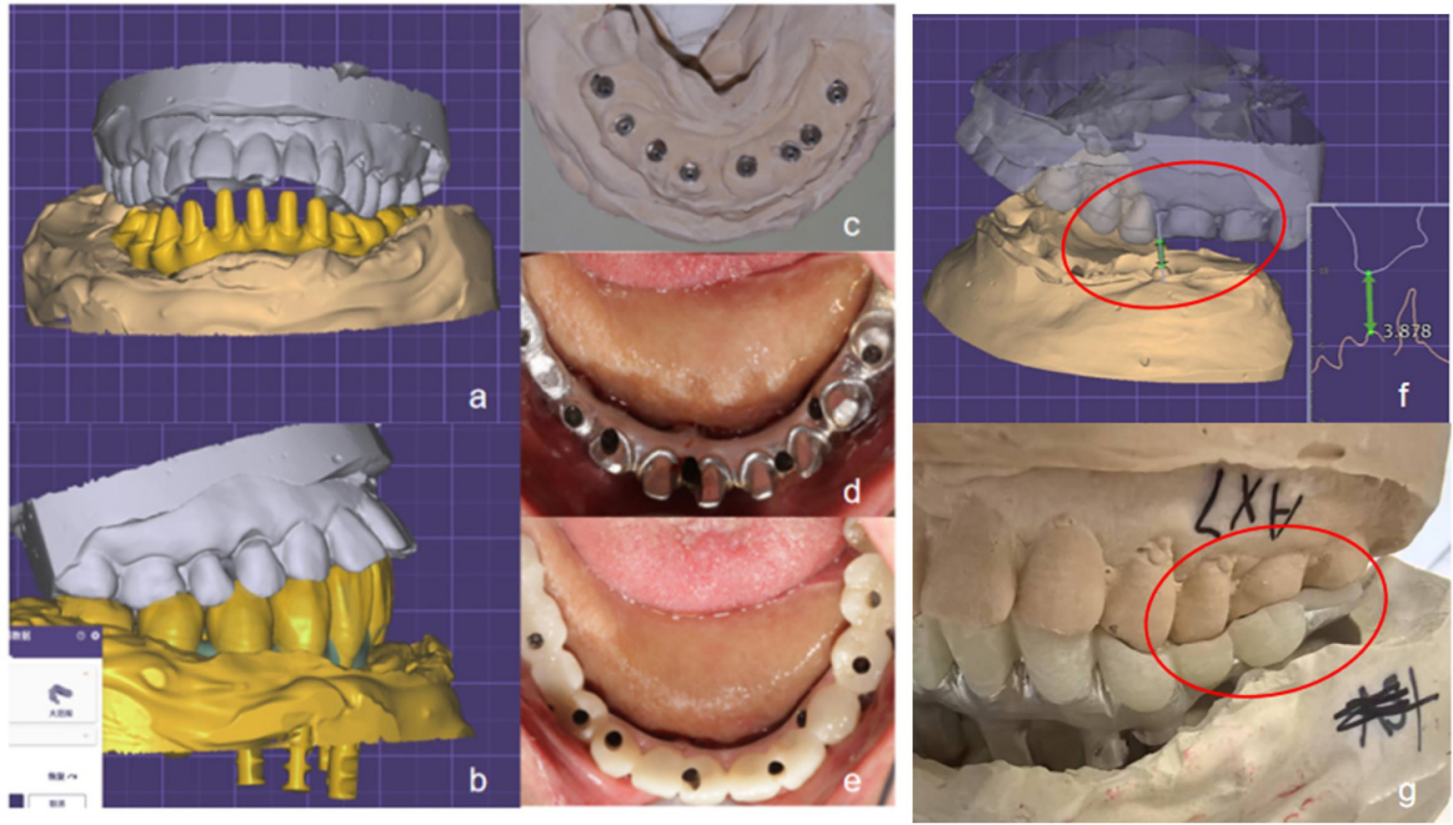
Digital impression taking. **(a,b)** Digital impression and virtual model; **(c)** Fabrication of the master cast; **(d,e)** Intraoral try-in of the provisional restoration.

**Figure 6:**
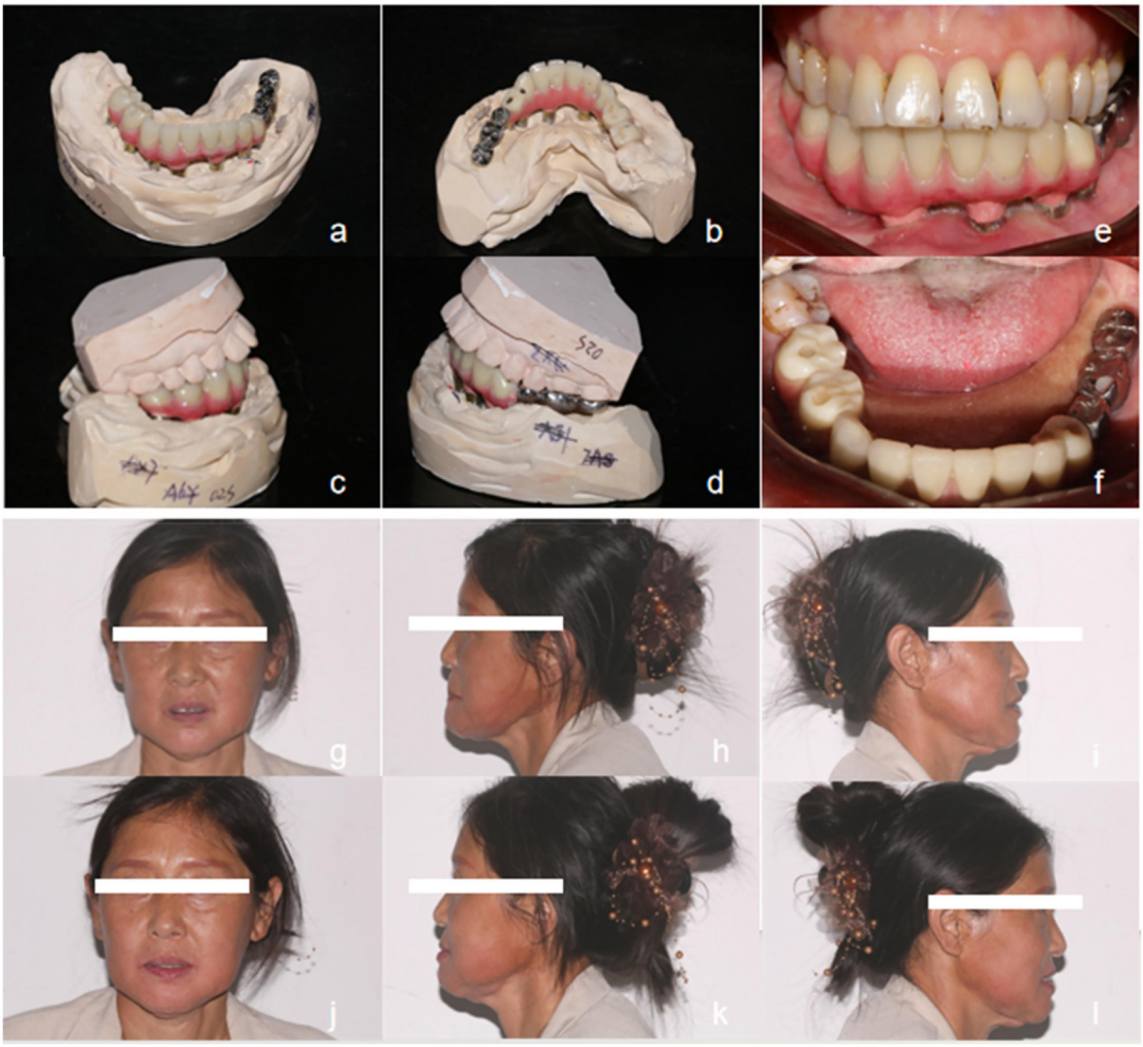
Denture fitting. **(a–d)** Definitive prosthesis laboratory view; **(e,f)** Intraoral view after delivery of the final prosthesis; **(h–i)** Frontal view of the seated restoration; **(j–l)** Lateral view of the prosthesis *in situ*.

At the 15-day follow-up appointment following the second-stage surgery, the soft tissues had healed satisfactorily. A digital impression was acquired, and the definitive prosthesis was fabricated (Figures 5a–c). Two weeks later, a try-in of the provisional resin prosthesis was performed, and occlusal adjustments were made to achieve proper vertical dimension and functional harmony (Figures 5d,e). The final restoration—a pure titanium framework supporting zirconia crowns and pure titanium prosthetic components—was subsequently delivered, completing the implant-supported superstructure. Following prosthesis placement, the patient exhibited good occlusal function and adaptation. Intraoral examination showed no signs of inflammation, swelling, or discomfort in the peri-implant soft tissues. An improvement in facial contour was also noted post-restoration (Figure 6).

Although metal-ceramic crowns and zirconia all-ceramic crowns are commonly used in clinical practice for supra-structure prostheses due to their aesthetic appeal and good biocompatibility, ceramic materials are inherently brittle. Fractures of these materials can compromise the success of implant-supported restorations. Additionally, such restorations require adequate interocclusal space and are not suitable for patients with excessively tight occlusion. In this case, the patient presented with tight occlusion on the left side of the mandible and maxilla, and the available interocclusal space was insufficient for a zirconia crown. The intraoral occlusal relationship is illustrated in Figures 5f, g. From a biomechanical perspective, the magnitude, direction, and location of loading are critical factors influencing stress distribution. The occlusal pattern and force exerted on implant-supported restorations can affect their long-term survival. According to Ercal (4), the peak equivalent stress on the implant and surrounding bone tissue does not significantly differ with the use of different supra-structure materials. Moreover, supra-structures with lower or higher elastic moduli do not markedly alter the stress distribution in the implant and bone. Studies have shown that there is no positive correlation between the choice of restorative material and increased stress in bone tissue (5). Therefore, to adhere to the principle of “no contact under light biting and minimal contact under heavy biting” for implant-supported crowns, a pure titanium crown was selected for the mandibular posterior tooth. The patient consented to this treatment plan.

#### 2.3.5 Maintenance after planting restoration

One to two weeks after prosthesis delivery: The patient was instructed to rinse with 0.12% chlorhexidine gluconate mouthwash and to clean the flap-graft region using a soft-bristled brush with a head 30% smaller than conventional sizes to avoid mechanical trauma. Starting one month after prosthesis delivery: The patient was advised to incorporate a 0.8-mm interdental brush for daily cleaning of the sulcus around the abutments, with particular emphasis on plaque control in the flap fold area. At each three-month follow-up visit: Soft tissue recession was measured using a periodontal probe (all measurements <0.5 mm), and professional polishing was performed around the abutments and prosthesis. During the follow-up visit, the patient reported no significant discomfort. The implant-supported crown exhibited satisfactory contour and proper occlusal relationship. The patient expressed satisfaction with both the aesthetic outcome and the restoration of occlusal function. An intraoral rinse and cleaning were performed during the appointment. The patient was advised to maintain good oral hygiene and return for regular follow-up observations.

## 3 Discussion

### 3.1 Bone grafting combined with implant placement

The free vascularized fibula flap has become one of the preferred approaches for mandibular reconstruction due to its favorable anatomical characteristics, which include a bicortical structure resembling the native mandible, adequate bone length and width, rich vascular supply, and high bone density (6, 7). Studies indicate that there are no significant differences in implant survival or postoperative infection rates between immediate and delayed implant placement following fibula flap reconstruction (8). Immediate implantation offers several advantages, including a reduction in the number of surgical interventions, decreased overall treatment costs, and earlier restoration of masticatory function. However, this approach is associated with longer operative times, increased technical complexity, and a potentially higher risk of systemic complications.In contrast, delayed implantation allows for a more thorough evaluation of bone and soft tissue healing, flap viability, and functional adaptation, facilitating more deliberate and individualized implant positioning and prosthetically-driven planning. The main drawbacks of this strategy include an extended overall treatment timeline and delayed rehabilitation of occlusal function (9, 10).

The decision to remove a reconstruction plate following jaw reconstruction is typically reserved for patients with significant clinical symptoms, such as local purulent discharge, plate exposure, fracture, loosening, or the need for subsequent implant placement. Conversely, elective removal is not generally recommended for asymptomatic elderly patients. In the present case, the patient presented with a large composite defect involving both the anterior and posterior mandible and an unstable occlusal relationship. The titanium plate, which had been *in situ* for nine years, remained well-fixed. It did not interfere with dental implant placement or compromise the long-term prosthetic outcome. Furthermore, the plate and screws provided a secondary stabilizing effect alongside the implants. Given these factors and the patient's own reluctance to undergo a second surgery for plate removal, the decision was made to retain the hardware.

### 3.2 Soft tissue management

While bone defect dimensions can be reasonably estimated during mandibular reconstruction with a fibula flap, soft tissue changes remain challenging to anticipate. Excess flap thickness may compromise the peri-implant mucosal seal, resulting in a shallow vestibular depth and excessive transmucosal emergence profile of the implant. Furthermore, redundant “skin paddle” tissue around the implant can foster an environment prone to peri-implant inflammation. The persistent traction from the soft tissue paddle of a free fibula flap may compromise the seal of the implant mucosal cuff, leading to plaque accumulation and impairing peri-implant hygiene. The transmucosal emergence area of the restoration at the mucosal margin represents a critical interface where plaque first contacts the peri-implant soft tissues. Inadequate plaque control further increases the risk of peri-implantitis (11). Study findings indicate that excessive vertical soft tissue thickness has a significant adverse effect on peri-implant tissue health (12). To mitigate the risk of peri-implantitis, the following measures can be adopted: (1) During benign tumor resection, preserve as much keratinized gingiva as possible to provide favorable soft tissue conditions for subsequent implant restoration. (2) When performing flap transplantation, prioritize a fibula fascial flap without a skin paddle to avoid the influence of excessive paddle thickness on future prosthetics. (3) Remove redundant “paddle” tissue from the flap during the first-stage surgery. (4) Restore the oral vestibular sulcus morphology surgically and, if necessary, utilize palatal keratinized mucosa grafts to reconstruct keratinized gingiva for improved sealing around implants. However, such additional procedures may increase trauma, complicate rehabilitation, and raise treatment costs. (5) Utilize customized abutments to establish favorable soft tissue architecture. Compared to other soft tissue management approaches, this method is relatively straightforward and effective, though its efficacy may be limited in cases with deep transmucosal emergence profiles. (6) Enhance adherence to standardized clinical protocols and reinforce postoperative oral hygiene education for patients. The management of peri-implant soft tissues aims to facilitate the development and maintenance of healthy peri-implant soft tissue while meeting patients’ aesthetic expectations, with an emphasis on minimizing the number of surgical interventions (13).

#### 3.2.1 Layered fat removal and gingival flap formation

In contrast to conventional approaches that either preserve the entire skin flap or perform aggressive thinning, this case utilized a refined soft tissue technique involving selective debulking of the superficial adipose layer while preserving the deep fat containing the vital vascular network. This method effectively reduces soft tissue bulk without compromising flap perfusion. Wang et al. similarly reported improvements in free fibula flap design by partial removal of the skin paddle to reduce soft tissue thickness (14). In addition to layered fat reduction, the use of a palatal keratinized mucosa graft can help reconstruct keratinized gingiva and establish a stable peri-implant seal. Zhang et al. demonstrated that implants with palatal mucosal grafts exhibited significantly less marginal bone loss compared to non-grafted sites, which may be attributed to earlier functional loading and favorable biomechanical conditions (15, 16). Kumar et al.. proposed subperiosteal dissection with denture-guided epithelial regeneration (SD-DGER) as a predictable technique for managing peri-implant soft tissues in patients undergoing mandibular reconstruction (17). Peri-implant soft tissue management remains a critical determinant of long-term success in mandibular reconstruction patients. Although multiple techniques have been introduced, limitations such as technical complexity, donor site morbidity, and variable clinical outcomes hinder their widespread adoption. Thus, continuous refinement of techniques and accumulation of clinical experience are essential to optimize functional and aesthetic outcomes.

#### 3.2.2 Use of custom healing abutments

Healing abutments play a dual role in implant therapy. Primarily, they facilitate the healing of peri-implant soft and hard tissues during the postoperative phase, help maintain the natural soft tissue architecture, and establish an optimal gingival emergence profile for the definitive prosthesis. Secondly, they prevent the accumulation of plaque and debris around the implant platform, thereby reducing the risk of peri-implant inflammation (18). In the present case, anatomical discrepancies between the fibular graft and the native mandible resulted in significant vertical height differences and excessive thickness of the fibular muscle flap. Under these conditions, standard healing abutments may lead to complications such as inadequate transmucosal emergence or increased plaque retention. To address this, a dynamic assessment of soft tissue thickness was performed, and customized healing abutments were utilized to achieve improved adaptation and morphological guidance, thereby promoting the formation of a stable gingival cuff (19). While personalized healing abutments help preserve peri-implant soft tissue structure, careful consideration must be given to their material properties. Materials such as polyetheretherketone (PEEK) and resin require evaluation to avoid potential adverse effects—including bone resorption or soft tissue recession—during the critical healing phase. Studies suggest that the integration of CAD/CAM technology offers a promising avenue for efficiently fabricating patient-specific healing abutments that support alveolar ridge stability and enhance biological outcomes (20).

#### 3.2.3 Targeted oral hygiene maintenance

The soft tissues around the implant, particularly those derived from a fibular muscle flap, often demonstrate reduced elasticity, increased susceptibility to plaque accumulation, and inadequate self-cleaning capabilities compared to natural gingiva. These characteristics—thickened and lax tissue morphology—predispose the peri-implant region to inflammatory responses and ultimately elevate the risk of peri-implantitis (21). Therefore, systematic postoperative maintenance is essential for effective soft tissue management. Patients should be instructed to clean the flap area using a soft-bristled toothbrush specifically designed to minimize mechanical irritation. Additionally, the use of interdental brushes is recommended to access and remove plaque from the sulcular areas and flap folds between the abutment and soft tissue. Beyond daily home care, patients require regular clinical follow-ups for comprehensive monitoring and maintenance of peri-implant tissues after delivery of the final prosthesis to enable early detection and prevention of peri-implant pathologies. As illustrated in this case, delayed implant restoration following fibular flap reconstruction represents a critical approach for rehabilitating extensive mandibular defects and restoring masticatory function. Successful implant therapy necessitates an integrated approach to soft tissue management. This requires coordinated efforts across all phases—from meticulous treatment planning and precise clinical execution by the clinician to consistent follow-up and diligent oral hygiene maintenance by the patient—to collectively prevent soft tissue complications, thereby mitigating the risks of peri-implant mucositis and peri-implantitis. Nevertheless, the procedure demands considerable surgical expertise and entails significant technical challenges. It is therefore imperative to continually refine clinical protocols, develop scientifically sound and prosthetically-driven treatment plans, and implement rigorous long-term maintenance strategies to reduce postoperative complications and ensure sustainable outcomes.

## Data Availability

The original contributions presented in the study are included in the article/Supplementary Material, further inquiries can be directed to the corresponding authors.
